# Contrast Administration Impacts CT-Based Radiomics of Colorectal Liver Metastases and Non-Tumoral Liver Parenchyma Revealing the “Radiological” Tumour Microenvironment

**DOI:** 10.3390/diagnostics11071162

**Published:** 2021-06-25

**Authors:** Francesco Fiz, Guido Costa, Nicolò Gennaro, Ludovico la Bella, Alexandra Boichuk, Martina Sollini, Letterio S. Politi, Luca Balzarini, Guido Torzilli, Arturo Chiti, Luca Viganò

**Affiliations:** 1 Nuclear Medicine Unit, IRCCS Humanitas Research Hospital, Rozzano, 20089 Milan, Italy; martina.sollini@cancercenter.humanitas.it (M.S.); arturo.chiti@hunimed.eu (A.C.); 2 Division of Hepatobiliary and General Surgery, Department of Surgery, IRCCS Humanitas Research Hospital, Rozzano, 20089 Milan, Italy; guido.costa@humanitas.it (G.C.); guido.torzilli@hunimed.eu (G.T.); 3 Department of Diagnostic Imaging, IRCCS Humanitas Research Hospital, Rozzano, 20089 Milan, Italy; nicolo.gennaro@st.hunimed.eu (N.G.); letterio.politi@hunimed.eu (L.S.P.); luca.balzarini@humanitas.it (L.B.); 4 Department of Biomedical Sciences, Humanitas University, Pieve Emanuele, 20089 Milan, Italy; ludovico.labella@st.hunimed.eu (L.l.B.); alexandra.boichuk@st.hunimed.eu (A.B.)

**Keywords:** computed tomography, radiomics, texture analysis, colorectal liver metastases, contrast medium

## Abstract

The impact of the contrast medium on the radiomic textural features (TF) extracted from the CT scan is unclear. We investigated the modification of TFs of colorectal liver metastases (CLM), peritumoral tissue, and liver parenchyma. One hundred and sixty-two patients with 409 CLMs undergoing resection (2017–2020) into a single institution were considered. We analyzed the following volumes of interest (VOIs): The CLM (Tumor-VOI); a 5-mm parenchyma rim around the CLM (Margin-VOI); and a 2-mL sample of parenchyma distant from CLM (Liver-VOI). Forty-five TFs were extracted from each VOI (LIFEx^®®^). Contrast enhancement affected most TFs of the Tumor-VOI (71%) and Margin-VOI (62%), and part of those of the Liver-VOI (44%, *p* = 0.010). After contrast administration, entropy increased and energy decreased in the Tumor-VOI (0.93 ± 0.10 vs. 0.85 ± 0.14 in pre-contrast; 0.14 ± 0.03 vs. 0.18 ± 0.04, *p* < 0.001) and Margin-VOI (0.89 ± 0.11 vs. 0.85 ± 0.12; 0.16 ± 0.04 vs. 0.18 ± 0.04, *p* < 0.001), while remaining stable in the Liver-VOI. Comparing the VOIs, pre-contrast Tumor and Margin-VOI had similar entropy and energy (0.85/0.18 for both), while Liver-VOI had lower values (0.76/0.21, *p* < 0.001). In the portal phase, a gradient was observed (entropy: Tumor > Margin > Liver; energy: Tumor < Margin < Liver, *p* < 0.001). Contrast enhancement affected TFs of CLM, while it did not modify entropy and energy of parenchyma. TFs of the peritumoral tissue had modifications similar to the Tumor-VOI despite its radiological aspect being equal to non-tumoral parenchyma.

## 1. Introduction

The liver is a frequent target for solid tumors’ metastases, especially from colorectal cancer [1,2]. Aggressive systemic therapies are administered, aiming at transforming cancer into a chronic disease [2]. In selected patients, loco-regional therapies, namely surgery or ablation, can improve prognosis [3,4,5]. Imaging plays a pivotal role in clinical decisions [6,7], but a precision medicine approach should consider tumor biology rather than just morphology to plan optimal management of patients. This is an unmet need in current oncological practice since available biomarkers do not predict survival consistently or can be identified on the surgical specimen only a posteriori [7,8,9,10].

In recent years, the textural analysis of radiological images, named “radiomics”, has attracted considerable interest [11,12]. This method enables the identification of voxel patterns that correlate with the biological properties of the analyzed tissue, such as vascularization and proliferation indices. Radiomic analyses demonstrated good performances in the non-invasive prediction of pathology data, effectiveness of systemic therapies, and survival in several diseases [13,14,15]. Despite these encouraging results, radiomics are not embedded in current clinical practice yet. The main limitations are lack of interpretability of radiomic features, unavailability of reliable cut-off values, and heterogeneity of analyses in term of software parameters and imaging phases. Most evidence in radiomics of liver metastases is based on the analysis of the portal venous phase of computed tomography (CT) [15]. However, the impact of CT contrast medium administration on radiomic parameters is still unclear and there is no robust evidence that the portal phase is the best dataset for performing texture analysis. Moreover, CT portal phase scan suffers from a wide heterogeneity related to acquisition protocols and patients’ characteristics. Conversely, pre-contrast images are much more standardized and may provide textural information complementary to or even clinically more relevant than that of portal images.

The present study was designed to assess the impact of contrast medium on the radiomic analyses of three different biological contexts: The tumor, the peritumoral microenvironment, and the normal liver parenchyma. The parenchyma surrounding colorectal liver metastases appears as the normal liver at standard radiology, but harbors relevant biomarkers, such as immune infiltrate and microsatellites [16,17,18]. In detail, we aimed to clarify which textural parameters of the tumor, the peritumoral tissue, and the non-tumoral parenchyma are modified by the contrast enhancement, and to compare the radiomic characteristics of the three tissues before and after the contrast medium administration.

## 2. Materials and Methods

### 2.1. Study Population

We retrospectively considered all consecutive patients that underwent liver resection for colorectal liver metastases (CLM) between January 2017 and July 2020. Only patients with a contrast-enhanced abdominal CT scan performed at our institution before surgery according to standardized protocols and with subsequent histological confirmation of CLM were included. We used the following additional inclusion criteria: Age ≥ 18 years; at least one CLM detectable at CT scan with diameter ≥ 10 mm; and availability of multiphase CT scan for review. We adopted the following exclusion criteria: Diagnosis of CLM not confirmed at final pathology; lack of an adequate portal phase of the CT scan; and movement or high-density material artefacts affecting the analysis. Lesions with a diameter < 10 mm were excluded from the study for the following reasons: They could not guarantee a sufficient number of voxels for the analysis (irregular shape); second-order radiomic features could be not reliable and/or informative in small lesions; and a 5-mm thick rim surrounding the metastasis was analyzed to evaluate the peritumoral tissue (see below, Image Analysis) and it is probably too large for millimetric metastases. The study was carried out following the declaration of Helsinki and its later amendments. The local review board approved the study protocol, and the need for informed consent was waived.

The study compared the radiomic features extracted from the pre-contrast and portal phases of preoperative CT scan. Features were extracted from (1) CLM, (2) peritumoral tissue, and (3) non-tumoral liver parenchyma remote from CLM. In the presence of multiple metastases, only nodules ≥ 10 mm and up to five CLMs per patient were analyzed. When multiple CLMs were considered, the peritumoral tissue of each CLM was analyzed. The analysis of non-tumoral parenchyma entailed the evaluation of a single volume of interest (VOI) per patient (see details below).

Two separate analyses were performed. First, radiomic data extracted from pre-contrast phase and portal phase were compared. We also analyzed the impact of tumor volume on the variation of radiomic features between the two phases. Secondly, we compared the radiomic features of CLM, peritumoral tissue, and remote non-tumoral parenchyma in the same phase, i.e., in the pre-contrast phase and the portal one.

### 2.2. Image Acquisition

Patients with CLM underwent a standardized CT protocol with multiphasic breath-hold acquisitions including a pre-contrast phase, an automatically bolus-triggered arterial phase, a portal phase (75 s—contrast administration delay), and an equilibrium (late) phase (3 min—contrast administration delay). Contrast administration (Iomeron 300 mg/mL; Bracco Diagnostics, Milan, Italy) was followed by a 30-mL saline flush (0.9%). As is typical for liver imaging, the dose of contrast agent was maximized, ranging 90–150 mL according to patients’ body weight. Bolus tracking over the abdominal aorta near the celiac axis (threshold at 120 Hounsfield units) was used to time the arterial acquisition. CT images were acquired on three different devices (Philips Healthliners, Amsterdam, The Netherlands and GE Healthcare, Wauwatosa (WI), USA) with a similar set-up in terms of detector collimation (0.625 mm), current (280–400 mA), tension (120–140 kV), and pitch (0.975). Automatic exposure control based on the X-ray attenuation on scout images was used.

### 2.3. Image Analysis

The LifeX 5.1 software (LITO, CEA, Inserm, CNRS, Univ. Paris-Sud, Université Paris Saclay) was used for texture analysis [19]. The segmentation was performed by a physician (FF), with ten-year experience in tumor imaging and segmentation. First, a VOI was manually drawn on the portal phase of the CT images to include the selected CLM entirely (Tumor VOI). All portions of the tumor evident in the portal phase images were included in the Tumor VOI; the peripheral ring of enhancement of metastases, if present, was also included in the Tumor VOI. Then, a VOI of peritumoral tissue was automatically generated by capturing a 5-mm thick rim surrounding the CLM VOI (Margin VOI). The margin VOI was reviewed to remove any portion of tissue other than liver parenchyma (e.g., lung parenchyma, gallbladder, large vessels, and colon) manually. Finally, a cylindrical VOI (radius 5 mm and height 25 mm; about 2 mL of tissue) was drawn on non-tumoral parenchyma remote from CLM (Liver VOI). The VOIs identified on the portal phase were then copied on the pre-contrast phase. Whenever needed, positioning of VOIs was manually adjusted. Textural analyses of each extracted VOI were performed separately.

### 2.4. Features Selection and Statistical Analyses

The software package automatically provided 68 variables. We excluded the indices identical in the two VOIs (e.g., volume-related variables, parameters describing the number of grey levels, bin size, distance of neighbors, and some other technical parameters) and indices related to the VOI layers (the number of layers could vary according to the VOI size). After this selection, 45 radiomic parameters were analyzed, including 7 conventional descriptors of the grey levels (HUmean, HUmin, HUmax, HUstd, and the HU tertiles), 6 first-order features, seven grey-level co-occurrence matrices (GLCM), 11 grey-level run length matrices (GLRLM), 3 neighboring grey-level difference matrices (NGLDM), and 11 grey-level zone length matrices (GLZLM).

Continuous variables were assessed graphically to determine distribution normality, and they were evaluated with parametric (unpaired *t*-test) or non-parametric (Mann-Whitney U-test) tests, accordingly. Categorical variables were compared with the Chi-square or Fisher’s exact tests, as appropriate. Bonferroni correction for multiple comparisons was applied: Whenever the 45 radiomic features were analyzed, a *p*-value ≤ 0.001 was considered significant. For the remaining analyses, a *p*-value < 0.05 was considered significant. To analyze the impact of volume of CLM on the variation of textural features between the pre-contrast phase and portal phase, we compared two groups of CLMs identified on the basis of the median value of volume. The analyses were carried out using SPSS V. 24 for Mac (IBM, Armonk, NY, USA) and STATA (StataCorp. 2019. Stata Statistical Software: Release 16. College Station, TX, USA: StataCorp LLC).

## 3. Results

**Figure 1:**
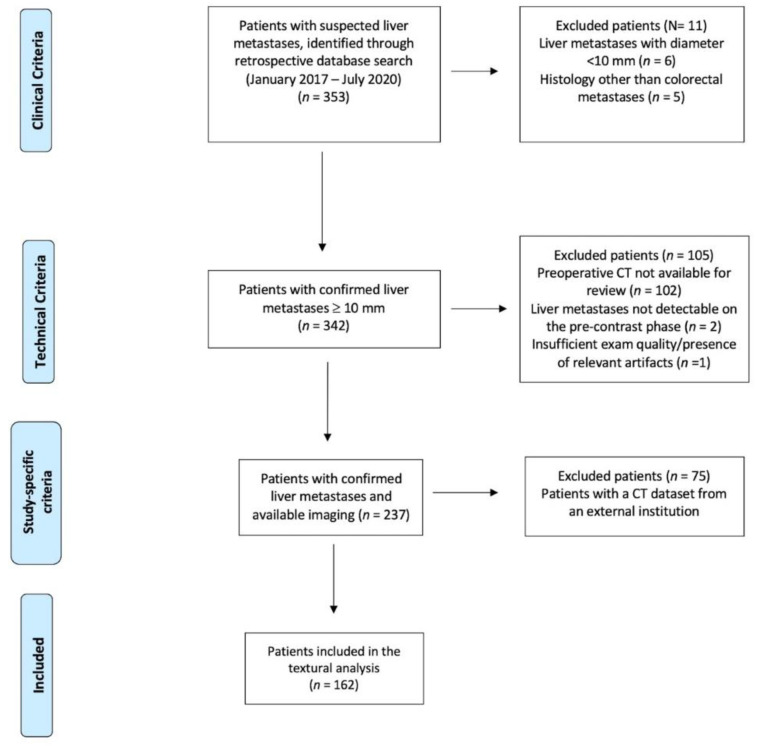
Patients’ inclusion flowchart.

A total of 409 CLMs were analyzed in 162 patients; see Table 1 for an overview of the patients’ characteristics and see Figure 1 for an outline of the included/excluded subjects.

Several radiomic features among the 45 extracted from each VOI had a significant variation after the administration of contrast medium, with a higher proportion in the Tumor VOI (variation of 32 features, 71%) and the Margin VOI (28, 62%) than in the Liver VOI (20, 44%, *p* = 0.010).

### 3.1. Radiomic Features of the Tumour VOI

**Figure 2:**
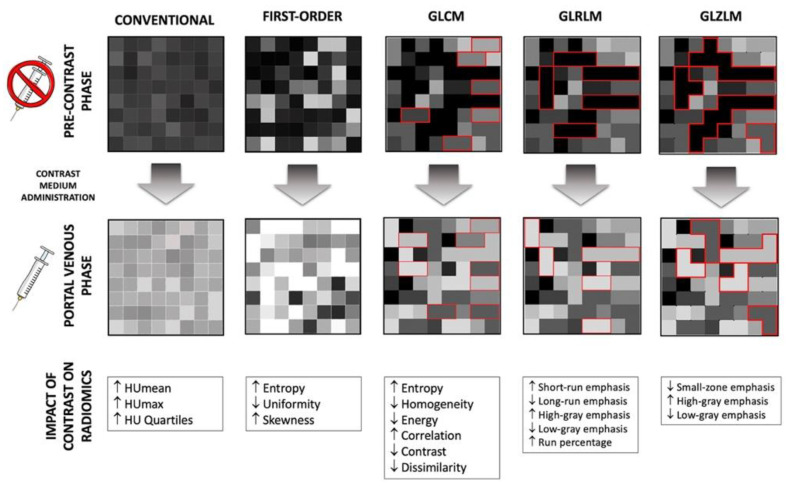
Effect of contrast medium on textural features in the Tumor-VOI. The modification of first-order and second-order features after contrast medium administration is detailed in white boxes. HU: Hounsfield Units; GLCM: Gray-level co-localization matrices; GLRLM: Gray-level run length matrices; GLZLM: Gray-level zone length matrices.

**Figure 3:**
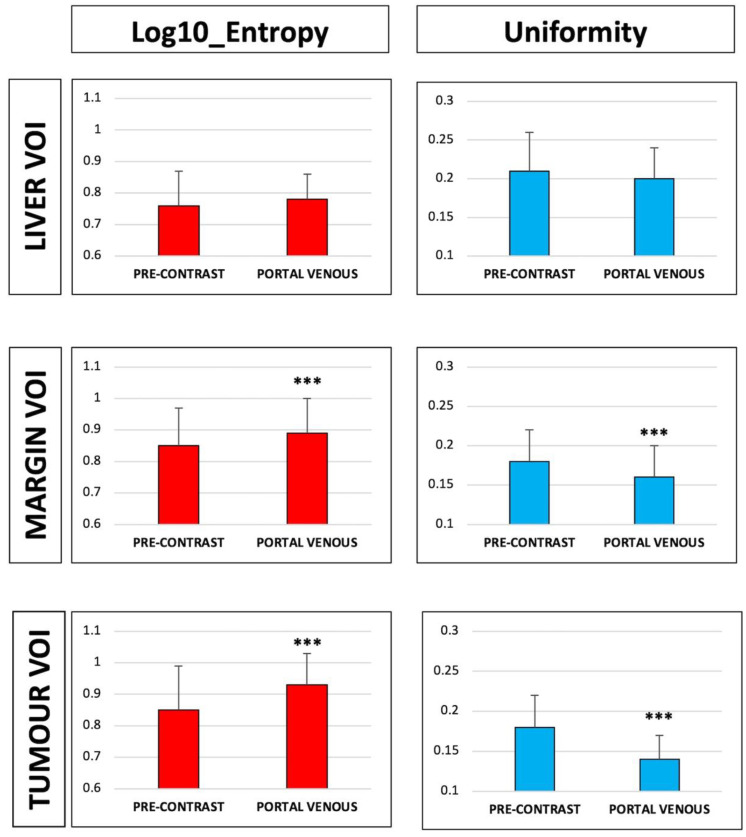
Comparison of entropy and uniformity of Tumor, Margin, and Liver volume of interest (VOI) in the pre-contrast vs. portal phase. *** *p* < 0.001.

All first-order features but kurtosis changed after contrast administration: Intensity and entropy increased (HUmean: 71 ± 25 vs. 39 ± 30 in the pre-contrast phase, *p* < 0.001; log10 entropy: 0.93 ± 0.10 vs. 0.85 ± 0.14, *p* < 0.001); uniformity decreased (0.14 ± 0.03 vs. 0.18 ± 0.04, *p* < 0.001); and the distribution of value became more left-skewed (0.14 ± 0.19 vs. −0.29 ± 2.08, *p* < 0.001). Of the second-order features, GLCM, GLRLM, and small zone-related ones changed after contrast. Table 2 and Figure 2 and Figure 3 summarize variations of radiomic features of the Tumor VOI after enhancement.

We investigated the impact of tumor volume on the variation of radiomic features. The median volume of CLMs was 4.1 mL. The large and low-volume groups included 205 (mean volume 20.1 ± 33.6 mL) and 204 (mean volume 1.1 ± 0.7 mL) CLMs, respectively. All first-order features and most second-order features had the same variation in the two groups (Supplementary Table S1).

### 3.2. Radiomic Features of the Margin VOI

All first-order features but skewness and kurtosis had a significant change in the portal phase as compared to the pre-contrast phase. The direction of variations was the same as that observed in Tumor VOI: Increase of intensity (103 ± 21 vs. 45 ± 23 in the pre-contrast phase, *p* < 0.001) and entropy (0.89 ± 0.11 vs. 0.85 ± 0.12, *p* < 0.001); and decrease of uniformity (0.16 ± 0.04 vs. 0.18 ± 0.04, *p* < 0.001).

Most second-order features varied as well, except for measures of non-uniformity of runs and zones. Table 3 and Figure 3 summarize variations of radiomic features of the Margin VOI after enhancement.

### 3.3. Radiomic Features of the Liver VOI

Of the first-order features, intensity increased (106 ± 20 vs. 54 ± 12 in the pre-contrast phase, *p* < 0.001), values were more left-skewed (0.24 ± 0.41 vs. −0.07 ± 0.63, *p* < 0.001), and kurtosis was flatter (3.73 ± 1.13 vs. 3.55 ± 4.41, *p* < 0.001) after the contrast medium administration. Uniformity and entropy had no significant variation between the two phases. Of the second-order features, GLCM ones, measures of non-uniformity of runs and zone, the level of spatial rate of change in intensity, and the measures of the homogeneity of the homogeneous runs and zones were similar in the two phases. Table 4 and Figure 3 summarize variations of radiomic features of the Liver VOI after enhancement.

### 3.4. Comparison of Radiomic Features across VOIs of the Same Series

We compared radiomic features of the three VOIs (Tumor, Margin, and Liver) in the pre-contrast and the portal phase. Considering intensity, Margin and Liver VOIs had similar values in the portal phase (104 ± 21 and 106 ± 20, respectively), higher than Tumor VOI (71 ± 25, *p* < 0.001).

**Figure 4:**
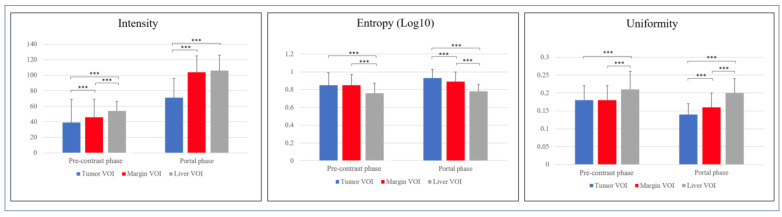
Comparison of intensity, entropy, and uniformity of Tumor volume of interest (VOI) vs. Margin VOI vs. Liver VOI in the pre-contrast and portal phase. *** *p* < 0.001.

**Figure 5:**
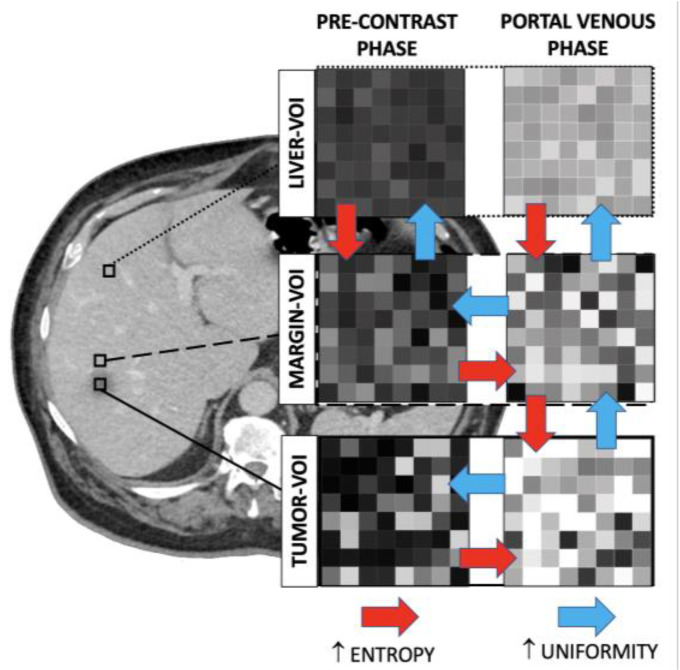
Entropy and uniformity across volumes of interests (VOIs) in the two phases. Red arrow: Significant increase in entropy; Blue arrow: Significant increase in uniformity.

Considering entropy, in the pre-contrast CT scans, Tumor and Margin VOIs had similar values (0.85 ± 0.14 and 0.85 ± 0.12), higher than Liver VOI (0.76 ± 0.11, *p* < 0.001 vs. both). After contrast enhancement, a gradient of entropy was evident, from Tumor VOI (0.93 ± 0.1) to Margin VOI (0.89 ± 0.11), and then to Liver VOI (0.78 ± 0.08, *p* < 0.001). Considering uniformity, in the pre-contrast scans, Tumor and Margin VOIs had similar values (0.18 ± 0.04 for both), lower than Liver VOI (0.21 ± 0.05), while the three VOIs had different values in the portal phase (0.14 ± 0.03, 0.16 ± 0.04, and 0.2 ± 0.04 for Tumor, Margin, and Liver VOI, respectively, *p* < 0.001). Variation of intensity, entropy, and homogeneity are summarized in Supplementary Table S2 and Figure 4 and Figure 5. The three VOIs had different kurtosis in both phases, while Tumor and Liver VOIs had similar skewness both in the basal and in the portal phase, more positive than that of Margin VOI. Second-order features were different among the three VOIs in the two phases, with few exceptions mainly concerning short-zones emphasis features (Supplementary Table S3).

## 4. Discussion

The present study demonstrated that contrast enhancement affects most textural features of colorectal liver metastases, while it does not modify entropy and energy of liver parenchyma. Radiomics of the peritumoral tissue had modifications similar to the tumor despite its radiological aspect equal to the non-tumoral liver parenchyma.

We analyzed the impact of contrast administration on radiomic parameters of liver parenchyma and liver tumors, specifically CLM. Some previous reports already studied the modification of first-order features of non-tumoral liver parenchyma after enhancement, demonstrating a significant variation in entropy, uniformity, skewness, and kurtosis [20,21]. However, those studies included a limited number of cases and considered dynamic contrast series. We collected a large cohort of patients and compared the pre-contrast and the portal phase of CT scan of the tumor, the liver parenchyma remote from CLM, and the peritumoral tissue. The latter was analyzed to depict the liver–tumor interface that harbors relevant biomarkers [16,17,18].

As expected, the contrast medium had a significant impact on tumor radiomics. It not only led to increasing of density-related features, which is already evident in standard radiology, but also influenced indicators of the curve shape (skewness) and the information content (uniformity and entropy). Considering second-order radiomic features, contrast enhancement impacted GLRLM and the small zone emphasis of GLZLM, which are related to microvasculature [22,23]. The radiomic analysis of portal phase provided a deeper insight on tumor heterogeneity and structural disorder than the analysis of pre-contrast series, as occurs in standard radiology. In fact, studies analyzing the portal venous phase of CT identified several associations between radiomics and tumor biology and prognosis [15]. It is noteworthy that the modification of radiomic features after contrast administration is independent of the tumor size.

In the present series, the potentialities of radiomics were clearly evident for non-tumoral VOIs, i.e., the peritumoral tissue (Margin) and the healthy liver parenchyma distant from the CLM (Liver VOI). The two volumes had similar intensity, higher than the tumor. This feature reflects their similarity at the standard radiology visualization. However, they showed significant differences at radiomic evaluation. Considering the pre-contrast phase, entropy and heterogeneity of the Margin VOI were similar to those of the Tumor VOI, much higher than the normal parenchyma. After contrast administration, the peritumoral area behaved as the tumor, with entropy and heterogeneity increase, while the remote parenchyma had no modification of these two radiomic features. In the portal phase, those indices displayed a gradient from the tumor to the normal liver parenchyma, the peritumoral tissue having intermediate values. These results suggest that the peritumoral tissue is a transition area between the tumor and the healthy parenchyma, whose nature can be captured by texture analysis, while it is missed at standard qualitative evaluation. The radiomic analyses unveil this discrepancy even before contrast enhancement. We want to emphasize that the Margin VOI was drawn on the portal venous phase images by including only normal liver parenchyma, externally to the macroscopically visible tumor area. Any peripheral rim enhancement of CLM was included in the Tumor VOI and not in the Margin VOI. The present capability of radiomics to explore invisible-to-eye features of normal tissue is in line with previous analyses demonstrating the possibility to predict metastases occurrence based on the radiomic features of radiologically normal liver parenchyma [24,25].

In current radiomics research, textural information is used for the construction of predictive models of biological features with the ultimate purpose of facilitating and empowering the clinical decision-making [11,12]. The present data have a clinical relevance because they elucidated some key points that may address future research about radiomics of liver tumor and parenchyma. First, even if the contrast medium administration modified many radiomic parameters, this was not true for all features. For instance, entropy and homogeneity of normal liver parenchyma remained stable in the two phases. Selected radiomics could be extracted from the pre-contrast phase of CT. Second, as expected, radiomic analysis of contrast-enhanced images provided richer information and granted better stratification of tissues than that of pre-contrast ones, especially for CLM and the peritumoral area. Most studies concerning textural analyses of CLM focused on portal phase of CT scan, reproducing their practice in standard radiology [15]. It is not by chance that they identified clinically relevant radiomic biomarkers in that phase. Nevertheless, pre-contrast and portal phases provided complementary data. Predictive models perform better when they consider both the enhanced and pre-contrast features than when they take into account only the portal phase [26,27]. This could not only be due to the availability of a larger quantity of data, but also because of the stability of the pre-contrast dataset [28]. Third, we highlighted a limited impact of tumor size on radiomics variation. This is extremely important when neoplasms have a wide range in size. Moreover, our study confirmed that texture analysis identifies peritumoral microenvironment as a separate entity, different from the normal liver parenchyma. Even if pathology data of peritumoral area are still to be explored, the radiomic analysis of the liver–tumor interface could provide relevant biomarkers [16,17,18].

Even if highly informative, radiomics extracted from contrast-enhanced phases appear to be conditioned by a wide variability related to acquisition parameters, technical factors, and patients’ characteristics. In our opinion, this does not undermine the potential contribution of texture analysis to non-invasive evaluation of tumor biology. The standardization of acquisition techniques is crucial [23,28,29], and harmonization protocols are pivotal to this aim [30,31]. Moreover, a sharp delineation between tumoral, peritumoral, and remote non-tumoral liver parenchyma was evident in both the pre-contrast and portal phases. The analysis of ratio between textural features of tumor and a reference VOI (e.g., normal parenchyma) could be an alternative approach to achieve standardization of data.

The present study has some limitations. It is a retrospective analysis, and CT data were acquired using different devices. However, CT images being collected from the same center had a standardized acquisition protocol and reconstruction parameters. A larger cohort of patients could show further dissimilarities of radiomic features. We collected a relevant number of patients with pathology confirmation of diagnosis. The present sample size guarantees the detection of all clinically relevant variations. Obtainable texture features are not theoretically limited to the ones described in the present work; in fact, some texture analysis methods can compute thousands of them [32,33]. We chose to extract and analyze the most standardized ones [34,35]. Analogously, the contrast-enhanced images also include the arterial and the equilibrium (late) phases. Because of the large amount of data to analyze (comparison of 45 textural features between two phases and among three tissues), we decided to focus on the portal venous phase images, which are the most used in texture analysis of liver metastases [15]. Finally, we did not analyze the association between radiomic features and pathology data and patients’ outcome. Even if of major interest, those topics were out of the scope of the present study and require dedicated analyses.

## 5. Conclusions

The administration of the CT contrast medium influenced most radiomic features of metastases, with modifications being independent of the tumor size. Oppositely, some textural parameters of liver parenchyma, namely homogeneity and entropy, were not modified by contrast enhancement and could be reliably assessed on pre-contrast CT scan. Peritumoral liver parenchyma had a specific radiomic pattern, more similar to CLM than to normal liver, further supporting the growing interest for this area as a niche of relevant biomarkers. Pre-contrast and portal phases provided complementary rather than overlapping data and should both be considered for their contribution to a precision medicine approach.

## Figures and Tables

**Table 1:** Characteristics of the studied population.

Patients’ Cohort (*n* = 162 Patients)	
Feature	Median (Range)-# (%)
Age (years)	62 (39–82)
Sex	
Male	100 (61.7%)
Female	62 (38.3%)
BMI (kg/m^2^)	25.4 (18.0–40.3)
Number of liver metastases per patient	2 (1–5)
Size of liver metastases (mm)	32 (10–71)
Preoperative chemotherapy	124 (76.5%)
>6 cycles	69 (42.6%)
Interval between CT and surgery (days)	23 (1–65)
**VOIs**	
Number of analyzed Tumor VOIs	409
Volume of metastases (mL, Tumor VOI)	4.1 (0.55–380)
Number of analyzed Margin VOIs	409
Volume of Margin VOI (mL)	9.6 (3.4–107.2)
Number of analyzed Liver VOIs	162
Volume of Liver VOI (mL)	1.96 (fixed value)

BMI: Body mass index; CT: Computed tomography; VOI: Volume of interest.

**Table 2:** Values of the radiomics features of Tumor volume of interest (VOI) in the portal and pre-contrast phases.

Class	Feature	Pre-Contrast Phase (Mean ± SD)	Portal Phase (Mean ± SD)	*p*	Class	Feature	Pre-Contrast Phase (Mean ± SD)	Portal Phase (Mean ± SD)	*p*
Conventional (Intensity)	MIN	−73 ± 171	−14 ± 94	<0.001	Gray-Level Run Length Matrices (GLRLM)	GLRLM_SRE	0.87 ± 0.03	0.89 ± 0.03	<0.001
	MEAN	39 ± 30	71 ± 25	<0.001		GLRLM_LRE	1.72 ± 0.28	1.64 ± 0.24	<0.001
	STD. DEVIATION	24 ± 32	22 ± 10	<0.001		GLRLM_LGRE	1.01 × 10^−4^ ± 7.89 × 10^−5^	866 × 10^−5^± 5.02 × 10^−6^	<0.001
	MAX	127 ± 133	163 ± 100	<0.001		GLRLM_HGRE	10,933.98 ± 515.11	11,619.98 ± 531.51	<0.001
	Q1	26 ± 51	57 ± 24	<0.001		GLRLM_SRLGE	8.90 × 10^−5^ ± 7.70 × 10^−5^	7.66 × 10^−5^± 4.79 × 10^−6^	<0.001
	Q2	41 ± 22	70 ± 25	<0.001		GLRLM_SRHGE	9558 ± 625	10,297 ± 678	<0.001
	Q3	53 ± 18	85 ± 26	<0.001		GLRLM_LRLGE	1.68 × 10^−4^ ± 9.21 × 10^−5^	1.43 × 10^−4^ ± 2.35 × 10^−5^	<0.001
First Order	HISTO_Skewness	−0.29 ± 2.08	0.14 ± 1.19	<0.001		GLRLM_LRHGE	18,797 ± 3034	18,998 ± 2652	0.317
	HISTO_Kurtosis	11.16 ± 28.13	5.85 ± 17.82	0.013		GLRLM_GLNU	704 ± 1422	855 ± 1868	0.197
	HISTO_Entropy_log10	0.85 ± 0.14	0.93 ± 0.10	<0.001		GLRLM_RLNU	3500 ± 6687	3696 ± 6933	0.684
	HISTO_Entropy_log2	2.83 ± 0.47	3.08 ± 0.34	<0.001		GLRLM_RP	0.84 ± 0.05	0.85 ± 0.04	<0.001
	HISTO_Energy (=Uniformity)	0.18 ± 0.04	0.14 ± 0.03	<0.001	Gray-Level Zone Length Matrices (GLZLM)	GLZLM_SZE	0.60 ± 0.06	0.58 ± 0.05	<0.001
Gray-Level Colocalization Matrices (GLCM)	GLCM_Homogeneity (=inverse difference)	0.51 ± 0.06	0.49 ± 0.05	<0.001		GLZLM_LZE	12,042 ± 29,885	9120 ± 30,290	0.005
	GLCM_Energy (=Angular second moment)	0.04 ± 0.02	0.03 ± 0.01	<0.001		GLZLM_LGZE	1.14 × 10^−4^ ± 2.12 × 10^−4^	8.61 × 10^−5^ ± 1.53 × 10^−5^	<0.001
	GLCM_Contrast (=Variance)	18.8 ± 180.71	6.24 ± 6.51	<0.001		GLZLM_HGZE	10,906 ± 763	11,779 ± 539	<0.001
	GLCM_Correlation	0.30 ± 0.15	0.37 ± 0.14	<0.001		GLZLM_SZLGE	7.47 × 10^−5^ ± 1.97 × 10^−4^	5.02 × 10^−5^ ± 1.21 × 10^−5^	<0.001
	GLCM_Entropy_log10	1.62 ± 0.23	1.73 ± 0.19	<0.001		GLZLM_SZHGE	6519 ± 680	6867 ± 642	<0.001
	GLCM_Entropy_log2(=Joint entropy)	5.37 ± 0.77	5.37 ± 0.77	<0.001		GLZLM_LZLGE	1.11 ± 2.74	0.80 ± 2.64	0.002
	GLCM_Dissimilarity	1.83 ± 2.69	1.78 ± 0.52	<0.001		GLZLM_LZHGE	1.31 × 10^8^ ± 3.26 × 10^−8^	1.04 × 10^8^ ± 3.49 × 10^−8^	0.011
NGLDM	NGLDM_Coarseness	8.47 ± 1.14	1.00 ± 1.49	0.350		GLZLM_GLNU	112.06 ± 63	58 ± 82.71	0.473
	NGLDM_Contrast	0.06 ± 0.11	0.06 ± 0.03	<0.001		GLZLM_ZLNU	218 ± 403	211 ± 336	0.783
	NGLDM_Busyness	0.34 ± 0.54	0.29 ± 0.46	0.266		GLZLM_ZP	0.14 ± 0.08	0.16 ± 0.08	0.004

SD: Standard deviation; Q1–3: Quartiles; NGLDM: Neighboring gray-level difference matrices; SRE/LRE/LGRE/HGRE: Short/long/high-gray/low-gray run emphasis; GL/RLNU: Gray-level/run length non-uniformity RP: Run percentage; SZE/LZE/LGZE/HGZE: Short/long/high-gray/low-gray zone emphasis; GL/ZLNU: Gray-level/zone length non-uniformity; ZP: Zone percentage.

**Table 3:** Values of the radiomics features of Margin volume of interest (VOI) in the portal and pre-contrast phases.

Class	Feature	Pre-Contrast Phase (Mean ± SD)	Portal Phase (Mean ± SD)	*p*	Class	Feature	Pre-Contrast Phase (Mean ± SD)	Portal Phase (Mean ± SD)	*p*
Conventional (Intensity)	MIN	−127 ± 232	−43 ± 162	<0.001	Gray-level run Length matrices (GLRLM)	GLRLM_SRE	0.87 ± 0.03	0.87 ± 0.03	0.023
	MEAN	45 ± 23	103 ± 21	<0.001		GLRLM_LRE	1.75 ± 0.23	1.72 ± 0.21	0.046
	STD. DEVIATION	27 ± 38	24 ± 23	<0.001		GLRLM_LGRE	1.05 × 10^−4^ ± 1.16 × 10^−4^	8.62 × 10^−5^ ± 7.46 × 10^−5^	<0.001
	MAX	136 ± 171	206 ± 167	<0.001		GLRLM_HGRE	11,067.4 ± 378.82	12,307.37 ± 440.84	<0.001
	Q1	36 ± 33	93 ± 20	<0.001		GLRLM_SRLGE	9.21 × 10^−5^ ± 1.08 × 10^−4^	7.56 × 10^−5^ ± 6.85 × 10^−5^	<0.001
	Q2	49 ± 11	105 ± 19	<0.001		GLRLM_SRHGE	9609.64 ± 454.57	10,745.25 ± 527.76	<0.001
	Q3	60 ± 11	117 ± 19	<0.001		GLRLM_LRLGE	1.76 × 10^−4^ ± 1.63 × 10^−4^	1.46 × 10^−4^± 1.10 × 10^−4^	<0.001
First Order	HISTO_Skewness	−1.00 ± 2.42	−0.68 ± 1.79	0.034		GLRLM_LRHGE	19,381.46 ± 2681.14	21,141.18 ± 2710.60	<0.001
	HISTO_Kurtosis	14.62 ± 29.94	10.94 ± 26.47	0.002		GLRLM_GLNU	1198.74 ± 1220.89	1061.56 ± 1051.90	0.089
	HISTO_Entropy_log10	0.85 ± 0.12	0.89 ± 0.11	<0.001		GLRLM_RLNU	5238.95 ± 5155.42	5329.76 ± 5355.55	0.807
	HISTO_Entropy_log2	2.83 ± 0.41	2.95 ± 0.35	<0.001		GLRLM_RP	0.83 ± 0.04	0.84 ± 0.03	0.025
	HISTO_Energy (=Uniformity)	0.18 ± 0.04	0.16 ± 0.04	<0.001	Gray-Level Zone Length Matrices (GLZLM)	GLZLM_SZE	0.61 ± 0.04	0.59 ± 0.03	<0.001
Gray-Level Colocalization Matrices (GLCM)	GLCM_Homogeneity (=inverse difference)	0.52 ± 0.05	0.51 ± 0.05	0.008		GLZLM_LZE	11,321.33 ± 15,687.66	8373.28 ± 10,870.41	0.001
	GLCM_Energy (=Angular second moment)	0.04 ± 0.02	0.03 ± 0.01	<0.001		GLZLM_LGZE	1.24 × 10^−4^ ± 1.93 × 10^−4^	9.26 × 10^−5^ ± 1.26 × 10^−4^	<0.001
	GLCM_Contrast (=Variance)	12.12 ± 54.31	6.21 ± 12.81	0.021		GLZLM_HGZE	10,901.23 ± 786.09	12,177.28 ± 616.16	<0.001
	GLCM_Correlation	0.34 ± 0.16	0.38 ± 0.12	<0.001		GLZLM_SZLGE	7.95 × 10^−5^ ± 1.39 × 10^−4^	5.56 × 10^−5^ ± 7.45 × 10^−5^	<0.001
	GLCM_Entropy_log10	1.63 ± 0.21	1.69 ± 1.18	<0.001		GLZLM_SZHGE	6576.90 ± 546.54	7209.92 ± 477.19	<0.001
	GLCM_Entropy_log2(=Joint entropy)	5.42 ± 0.68	5.63 ± 0.61	<0.001		GLZLM_LZLGE	1.02 ± 1.41	0.68 ± 0.89	<0.001
	GLCM_Dissimilarity	1.67 ± 1.01	1.63 ± 0.37	0.019		GLZLM_LZHGE	1.26 × 10^8^ ± 1.74 × 10^−8^	1.03 × 10^8^ ± 1.33 × 10^−8^	0.039
NGLDM	NGLDM_Coarseness	1.55 × 10^−3^± 1.23 × 10^−3^	1.79 × 10^−3^± 1.79 × 10^−3^	0.026		GLZLM_GLNU	105.01 ± 101.20	98.23 ± 94.25	0.327
	NGLDM_Contrast	0.04 ± 0.16	0.03 ± 0.02	0.039		GLZLM_ZLNU	366.34 ± 417.51	363.53 ± 387.52	0.921
	NGLDM_Busyness	0.46 ± 0.46	0.33 ± 0.27	<0.001		GLZLM_ZP	0.12 ± 0.05	0.13 ± 0.04	0.016

SD: Standard deviation; Q1–3: Quartiles; NGLDM: Neighboring gray-level difference matrices; SRE/LRE/LGRE/HGRE: Short/long/high-gray/low-gray run emphasis; GL/RLNU: Gray-level/run length non-uniformity RP: Run percentage; SZE/LZE/LGZE/HGZE: Short/long/high-gray/low-gray zone emphasis; GL/ZLNU: Gray-level/zone length non-uniformity; ZP: Zone percentage.

**Table 4:** Values of the radiomics features of Liver volume of interest (VOI) in the portal and pre-contrast phases.

Class	Feature	Pre-Contrast Phase (Mean ± SD)	Portal Phase (Mean ± SD)	*p*	CLASS	Class	Feature	Pre-Contrast Phase (Mean ± SD)	*p*
Conventional (Intensity)	MIN	−1 ± 77	59 ± 23	<0.001	Gray-level Run length Matrices (GLRLM)	GLRLM_SRE	0.85 ± 0.03	0.86 ± 0.03	0.602
	MEAN	54 ± 12	106 ± 20	<0.001		GLRLM_LRE	1.87 ± 0.28	1.85 ± 0.26	0.486
	STD. DEVIATION	15 ± 9	15 ± 3	0.011		GLRLM_LGRE	8.96 × 10^−5^ ± 5.79 × 10^−6^	8.11 × 10^−5^ ± 2.99 × 10^−6^	<0.001
	MAX	102 ± 25	167 ± 31	<0.001		GLRLM_HGRE	11,232 ± 257	12,364 ± 440	<0.001
	Q1	45 ± 12	96 ± 20	<0.001		GLRLM_SRLGE	7.65 × 10^−5^ ± 6.24 × 10^−6^	6.93 × 10^−5^ ± 3.41 × 10^−6^	<0.001
	Q2	54 ± 12	106± 20	<0.001		GLRLM_SRHGE	9586 ± 439	10,577 ± 541	<0.001
	Q3	64 ± 14	115 ± 20	<0.001		GLRLM_LRLGE	1.67 × 10^−4^ ± 2.64 × 10^−5^	1.50 × 10^−4^ ± 2.15 × 10^−5^	<0.001
First Order	HISTO_Skewness	−0.07 ± 0.63	0.24 ± 0.41	<0.001		GLRLM_LRHGE	20,976 ± 3240	22,812 ± 3252	<0.001
	HISTO_Kurtosis	3.55 ± 4.41	3.73 ± 1.13	<0.001		GLRLM_GLNU	188 ± 43	185 ± 41	0.506
	HISTO_Entropy_log10	0.76 ± 0.11	0.78 ± 0.08	0.201		GLRLM_RLNU	687 ± 211	703 ± 221	0.515
	HISTO_Entropy_log2	2.53 ± 0.35	2.57 ± 0.28	0.201		GLRLM_RP	0.81 ± 0.04	0.81 ± 0.04	0.551
	HISTO_ENERGY (=Uniformity)	0.21 ± 0.05	0.20 ± 0.04	0.230	Gray-Level Zone Length Matrices (GLZLM)	GLZLM_SZE	0.60 ± 0.04	0.59 ± 0.04	0.006
Gray-level Colocalization Matrices (GLCM)	GLCM_Homogeneity (=Inverse difference)	0.53 ± 0.06	0.53 ± 0.05	0.229		GLZLM_LZE	2700 ± 1929	2506 ± 1897	0.024
	GLCM_Energy (=Angular second moment)	0.05 ± 0.02	0.04 ± 0.02	0.109		GLZLM_LGZE	9.21 × 10^−5^ ± 3.57 × 10^−5^	8.09 × 10^−5^ ± 3.04 × 10^−6^	<0.001
	GLCM_Contrast (=Variance)	4.23 ± 7.32	3.71 ± 1.50	0.377		GLZLM_HGZE	11,222 ± 313	12,406 ± 456	<0.001
	GLCM_Correlation	0.17 ± 0.07	0.21 ± 0.08	<0.001		GLZLM_SZLGE	5.60 × 10^−5^ ± 2.90 × 10^−5^	4.77 × 10^−5^ ± 4.20 × 10^−6^	<0.001
	GLCM_Entropy_log10	1.50 ± 0.21	1.53 ± 0.17	0.135		GLZLM_SZHGE	6757 ± 471	7309 ± 591	<0.001
	GLCM_Entropy_log2 (=Joint entropy)	4.97 ± 0.68	5.08 ± 0.55	0.135		GLZLM_LZLGE	0.24 ± 0.17	0.20 ± 0.15	0.043
	GLCM_Dissimilarity	1.45 ± 0.58	1.45 ± 0.30	0.936		GLZLM_LZHGE	3.04 × 10^7^ ± 2.18 × 10^−7^	3.10 × 10^7^ ± 2.36 × 10^−7^	0.817
NGLDM	NGLDM_Coarseness	0.01 ± 0	0.01 ± 0	0.964		GLZLM_GLNU	17.42 ± 4.68	16.65 ± 4.83	0.151
	NGLDM_Contrast	0.05 ± 0.03	0.04 ± 0.01	0.009		GLZLM_ZLNU	43.68 ± 54.59	40.75 ± 22.18	0.531
	NGLDM_Busyness	0.15 ± 0.04	0.13 ± 0.04	<0.001		GLZLM_ZP	0.10 ± 0.05	0.10 ± 0.03	0.856

SD: Standard deviation; Q1–3: Quartiles; NGLDM: Neighboring gray level difference matrices; SRE/LRE/LGRE/HGRE: Short/long/high-gray/low-gray run emphasis; GL/RLNU: Gray-level/run length non-uniformity RP: Run percentage; SZE/LZE/LGZE/HGZE: Short/long/high-gray/low-gray zone emphasis; GL/ZLNU: Gray-level/zone length non-uniformity; ZP: Zone percentage.

## Data Availability

Data are available and can be obtained from the corresponding author upon reasonable request.

## Supplementary Materials

The following are available online at https://www.mdpi.com/article/10.3390/diagnostics11071162/s1, Table S1: Significance of feature difference of lesions’ high and volume VOIs between portal and pre-contrast phases, Table S2: Entropy and uniformity-related features values in the Tumor, Margin, and Liver VOI, Table S3: Differences in second-order features values across the Tumor, Margin, and Liver VOI in the portal and the pre-contrast phases.
