# Supplementary material for: Contrast Administration Impacts CT-Based Radiomics of Colorectal Liver Metastases and Non-Tumoral Liver Parenchyma Revealing the “Radiological” Tumour Microenvironment
Source: Diagnostics (Basel). 2021 Jun 25;11(7):1162. doi: 10.3390/diagnostics11071162 (PMC8305553; doi:10.3390/diagnostics11071162)
Supplement: Supplementary file 1 [file diagnostics-11-01162-s001.zip › diagnostics-1220105-supplementary.pdf]

Supplementary Tables

Table S1. Significance of feature difference of lesions' high and volume VOIs between portal and pre-contrast phases. Matching of variation was established by comparing the *p* values of the two groups. Bonferroni correction for multiple comparisons was applied: a *p*-value ≤ 0.001 was considered significant.

CLASS	FEATURE	*p* HIGH VOLUME	*p* LOW VOLUME	MATCH	CLASS	FEATURE	*p* HIGH VOLUME	*p* LOW VOLUME	MATCH
CONVENTIONAL (INTENSITY)	MIN	<0.001	<0.001	Y	GRAY LEVEL RUN-LENGTH MATRICES (GLRLM)	GLRLM_SRE	0.002	<0.001	N
	MEAN	<0.001	<0.001	Y		GLRLM_LRE	0.002	<0.001	N
	STD. DEVIATION	<0.001	<0.001	Y		GLRLM_LGRE	0.011	0.002	Y
	MAX	<0.001	<0.001	Y		GLRLM_HGRE	<0.001	<0.001	Y
	Q1	<0.001	<0.001	Y		GLRLM_SRLGE	0.018	0.011	Y
	Q2	<0.001	<0.001	Y		GLRLM_SRHGE	<0.001	<0.001	Y
	Q3	<0.001	<0.001	Y		GLRLM_LRLGE	<0.001	<0.001	Y
FIRST ORDER	HISTO_Skewness	<0.001	<0.001	Y		GLRLM_LRHGE	0.128	0.435	Y
	HISTO_Kurtosis	0.006	0.180	Y		GLRLM_GLNU	0.159	0.014	Y
	HISTO_Entropy_log10	<0.001	<0.001	Y		GLRLM_RLNU	0.717	0.554	Y
	HISTO_Entropy_log2	<0.001	<0.001	Y		GLRLM_RP	0.001	<0.001	Y
	HISTO_Energy (=Uniformity)	<0.001	<0.001	Y	GRAY LEVEL ZONE LENGTH MATRICES (GLZLM)	GLZLM_SZE	<0.001	0.007	N
GRAY LEVEL COLOCALIZATION MATRICES (GLCM)	GLCM_Homogeneity (=inverse difference)	<0.001	<0.001	Y		GLZLM_LZE	0.011	<0.001	N
	GLCM_Energy (=Angular second moment)	<0.001	<0.001	Y		GLZLM_LGZE	<0.001	<0.001	Y
	GLCM_Contrast (=Variance)	0.003	<0.001	N		GLZLM_HGZE	<0.001	<0.001	Y
	GLCM_Correlation	<0.001	<0.001	Y		GLZLM_SZLGE	<0.001	<0.001	Y
	GLCM_Entropy_log10	<0.001	<0.001	Y		GLZLM_SZHGE	<0.001	<0.001	Y
	GLCM_Entropy_log2(=Joint entropy)	<0.001	<0.001	Y		GLZLM_LZLGE	0.041	<0.001	N

	GLCM_Dissimilarity	0.001	<0.001	Y	GLZLM_LZHGE	0.026	<0.001	N
NGLDM	NGLDM_Coarseness	0.395	0.100	Y	GLZLM_GLNU	0.331	0.224	Y
	NGLDM_Contrast	<0.001	<0.001	Y	GLZLM_ZLNU	0.662	0.506	Y
	NGLDM_Busyness	0.387	0.008	Y	GLZLM_ZP	0.051	<0.001	N

Table S2. Entropy and uniformity-related features values in the tumour, Margin, and Liver VOI.

VARIABLE	HISTO_Entropy_log2	GLCM_Entropy_log2	HISTO_Energy (= uniformity)	GLCM_Homogeneity (=Inverse difference)	GLCM_Energy (=Angular second moment)	GLZLM_SZHGE
**PORTAL VENOUS PHASE**						
**TUMOR (MEAN ± SD)**	3.08 ± 0.34	5.76 ± 0.64	0.14 ± 0.03	0.49 ± 0.05	0.028 ± 0.014	6687 ± 642
**MARGIN (MEAN ± SD)**	2.95 ± 0.35	5.63 ± 0.61	0.16 ± 0.04	0.51± 0.05	0.033 ± 0.015	7210 ± 477
**LIVER (MEAN ± SD)**	2.57 ± 0.28	5.08 ± 0.55	0.2 ±0.04	0.53 ± 0.05	0.044 ± 0.018	7309± 591
**TUMOR VS MARGIN (*p*-VALUE)**	<0.001	0.002	<0.001	<0.001	<0.001	<0.001
**TUMOR VS LIVER (*p*-VALUE)**	<0.001	<0.001	<0.001	<0.001	<0.001	<0.001
**MARGIN VS LIVER (*p*-VALUE)**	<0.001	<0.001	<0.001	<0.001	<0.001	0.040
**PRE-CONTRAST PHASE**						
**TUMOR (MEAN ± SD)**	2.83 ± 0.47	5.37 ± 0.77	0.18 ± 0.04	0.51 ± 0.06	0.038 ± 0.017	6519 ± 680
**MARGIN (MEAN ± SD)**	2.83 ± 0.41	5.42 ± 0.68	0.18 ± 0.04	0.052 ± 0.05	0.037 ± 0.016	6577 ± 547
**LIVER (MEAN ± SD)**	2.53 ± 0.35	4.97 ± 0.68	0.21 ± 0.05	0.53 ± 0.06	0.048 ± 0.021	6757 ± 471

**TUMOR VS MARGIN (*p*-VALUE)**	0.979	0.375	0.432	0.077	0.810	0.185
**TUMOR VS LIVER (*p*-VALUE)**	<0.001	<0.001	<0.001	<0.001	<0.001	<0.001
**MARGIN VS LIVER (*p*-VALUE)**	<0.001	<0.001	<0.001	0.007	<0.001	<0.001

Table S3. Differences in second-order features values across the Tumour, Margin, and Liver VOI in the portal and the pre-contrast phases.

PHASE	PORTAL PHASE (*p* values)				PRE-CONTRAST PHASE (*p* values)			
**VARIABLE**	**GLCM_Homogeneity (=Inverse difference)**	**GLCM_Energy (=Angular second moment)**	**GLCM_Contrast (=Variance)**	**GLCM_Correlation**	**GLCM_Homogeneity (=Inverse difference)**	**GLCM_Energy (=Angular second moment)**	**GLCM_Contrast (=Variance)**	**GLCM_Correlation**
**TUMOR VS MARGIN**	<0.001	<0.001	0.013	6.50E-01	<0.001	<0.001	0.665	<0.001
**TUMOR VS LIVER**	<0.001	<0.001	<0.001	<0.001	<0.001	<0.001	<0.001	<0.001
**MARGIN VS LIVER**	<0.001	<0.001	<0.001	<0.001	<0.001	<0.001	<0.001	<0.001
**VARIABLE**	**GLCM_Entropy_log10**	**GLCM_Entropy_log2 (=Joint entropy)**	**GLCM_Dissimilarity**	**GLRLM_SRE**	**GLCM_Entropy_log10**	**GLCM_Entropy_log2 (=Joint entropy)**	**GLCM_Dissimilarity**	**GLRLM_SRE**
**TUMOR VS MARGIN**	0.02	0.02	<0.001	<0.001	0.375	0.375	0.292	0.021
**TUMOR VS LIVER**	<0.001	<0.001	<0.001	<0.001	<0.001	<0.001	<0.001	<0.001
**MARGIN VS LIVER**	<0.001	<0.001	<0.001	<0.001	<0.001	<0.001	0.009	<0.001
**VARIABLE**	**GLRLM_LRE**	**GLRLM_LGRE**	**GLRLM_HGRE**	**GLRLM_SRLGE**	**GLRLM_LRE**	**GLRLM_LGRE**	**GLRLM_HGRE**	**GLRLM_SRLGE**
**TUMOR VS MARGIN**	<0.001	<0.001	<0.001	<0.001	0.021	<0.001	<0.001	<0.001
**TUMOR VS LIVER**	<0.001	<0.001	<0.001	<0.001	<0.001	<0.001	<0.001	0.042
**MARGIN VS LIVER**	<0.001	0.03	0.082	<0.001	<0.001	<0.001	<0.001	<0.001
**VARIABLE**	**GLRLM_SRHGE**	**GLRLM_LRLGE**	**GLRLM_LRHGE**	**GLRLM_GLNU**	**GLRLM_SRHGE**	**GLRLM_LRLGE**	**GLRLM_LRHGE**	**GLRLM_GLNU**
**TUMOR VS MARGIN**	<0.001	0.545	<0.001	<0.001	0.0320	0.379	0.0039	0.0021
**TUMOR VS LIVER**	<0.001	0.001	<0.001	<0.001	0.6050	<0.001	<0.001	<0.001
**MARGIN VS LIVER**	<0.001	<0.001	<0.001	<0.001	0.5810	0.002	<0.001	<0.001
**VARIABLE**	**GLRLM_RLNU**	**GLRLM_RP**	**NGLDM_Coarseness**	**NGLDM_Contrast**	**GLRLM_RLNU**	**GLRLM_RP**	**NGLDM_Coarseness**	**NGLDM_Contrast**
**TUMOR VS MARGIN**	0.0020	<0.001	<0.001	<0.001	<0.001	0.017	<0.001	<0.001
**TUMOR VS LIVER**	<0.001	<0.001	0.002	<0.001	<0.001	<0.001	<0.001	0.226
**MARGIN VS LIVER**	<0.001	<0.001	<0.001	<0.001	<0.001	<0.001	<0.001	<0.001
**VARIABLE**	**NGLDM_Busyness**	**GLZLM_SZE**	**GLZLM_LZE**	**GLZLM_LGZE**	**NGLDM_Busyness**	**GLZLM_SZE**	**GLZLM_LZE**	**GLZLM_LGZE**
**TUMOR VS MARGIN**	<0.001	<0.001	<0.001	<0.001	<0.001	0.058	<0.001	0.002
**TUMOR VS LIVER**	<0.001	0.146	0.006	<0.001	<0.001	0.635	<0.001	<0.001
**MARGIN VS LIVER**	<0.001	0.143	<0.001	<0.001	<0.001	0.245	<0.001	0.04
**VARIABLE**	**GLZLM_HGZE**	**GLZLM_SZLGE**	**GLZLM_SZHGE**	**GLZLM_LZLGE**	**GLZLM_HGZE**	**GLZLM_SZLGE**	**GLZLM_SZHGE**	**GLZLM_LZLGE**

**TUMOR VS MARGIN**	<0.001	<0.001	<0.001	<0.001	<0.001	0.695	0.185	<0.001
**TUMOR VS LIVER**	<0.001	<0.001	<0.001	<0.001	<0.001	<0.001	<0.001	<0.001
**MARGIN VS LIVER**	<0.001	<0.001	0.039	<0.001	<0.001	0.035	<0.001	<0.001
**VARIABLE**	**GLZLM_LZHGE**	**GLZLM_GLNU**	**GLZLM_ZLNU**	**GLZLM_ZP**	**GLZLM_LZHGE**	**GLZLM_GLNU**	**GLZLM_ZLNU**	**GLZLM_ZP**
**TUMOR VS MARGIN**	<0.001	<0.001	<0.001	<0.001	<0.001	<0.001	<0.001	<0.001
**TUMOR VS LIVER**	0.009	<0.001	<0.001	<0.001	0.009	<0.001	<0.001	<0.001
**MARGIN VS LIVER**	<0.001	<0.001	<0.001	<0.001	<0.001	<0.001	<0.001	<0.001
